# When freedom of choice leads to bias: How threat fosters selective exposure to health information

**DOI:** 10.3389/fpsyg.2022.937699

**Published:** 2022-10-13

**Authors:** Oliver Wedderhoff, Anita Chasiotis, Tom Rosman

**Affiliations:** Research Literacy Unit, Leibniz Institute for Psychology, Trier, Germany

**Keywords:** selective exposure, health information, health threat, experimental study, information-seeking

## Abstract

Selective exposure to online health information can be ascribed to two related defense motives: the motivation to confirm one’s subjective perceptions and the motivation to protect relevant parts of the self-image, such as physical integrity. Our aim was to identify how these motives come into effect in the context of a health threat (fictitious feedback on an alleged heart disease risk). In a preregistered online study with *N* = 763 participants, we analyzed the impact of perceived and suggested risk on the degree of bias in selecting risk-related information on a fictitious Google search results page. Applying a 2 × 2 design with the experimental factor “risk feedback” and the quasi-experimental factor “perceived risk,” we formulated six hypotheses. First, we expected a main effect of perceived risk on selective exposure to information suggesting no risk, and second, we hypothesized a main effect of perceived risk on mean quality rating of information suggesting a risk. Third, we proposed a main effect of risk feedback on selective exposure to information which suggests no risk, and fourth, we proposed a main effect of risk feedback on mean quality rating of information suggesting a risk. Fifth, we expected an interaction effect between perceived and suggested risk, and sixth, we proposed an interaction effect between perceived and suggested risk in different forms for each of the four conditions on quality ratings. Only the third hypothesis was confirmed: Receiving information which suggested a health risk increased the tendency to select information denying the risk. Additional exploratory analyses revealed moderator effects of health information literacy and participant age on the aforementioned relationships. In sum, our results underline the crucial role of defense motives in the context of a suggested health threat.

## Introduction

Health information plays a major role in everyday life. It influences, for example, how you shape your nutrition, how and how often you brush your teeth, or the amount of sleep you try to get each night. It also helps you to recognize potential alarm symptoms, and it may shape your opinions on political agendas (e.g., on vaccination programs or on coronavirus quarantine regulations). Nowadays, vast amounts of health information are freely accessible through all kinds of information sources, most notably through the internet (Fox and Duggan, 2013). However, health information is often multifaceted, and health information sources vary considerably in their quality and scope. Therefore, the question of how and why humans consider specific information while rejecting other information is of utter importance to improve individual access to helpful, objective, and scientifically sound health information.

A number of explicit and implicit intentions shape health information seeking due to the self-responsibility of an independent information search and the peculiarities of the health domain, which, for example, can threaten psychological well-being as well as physical integrity. So-called defense motives are triggered in response to threatening information and lead to favoring and specifically searching for information corresponding to one’s self-image (Kunda, 1990; Olson and Stone, 2005; Sherman and Cohen, 2016). Sometimes, defense motives can also provoke a devaluation of non-conforming or threatening information (Ditto and Lopez, 1992; Edwards and Smith, 1996). These defensive mechanisms, which emerge as behavioral consequences from defense motives, oppose aspirations of a holistic, accurate, and complete search (Albarracin et al., 2005; Hart et al., 2009). Correspondingly, bias within the information selection, consideration, and evaluation process are observed in many studies (Schweiger et al., 2014; Greving and Sassenberg, 2015; Sassenberg and Greving, 2016). As threat plays a huge role in triggering defense motives, the present paper investigates the relationship between different types of health threats and the selection of health information. In order to induce threat, fictitious connections between a personality disposition and a health issue were suggested. In the literature, the phenomenon of a biased selection of information (primarily with a preference for non-threatening information that serves one’s self-image) is referred to by varying terminologies. In the present paper, we will use the term “selective exposure” (Frey, 1986) to indicate bias related to the selection and consideration of information, as we think it is best suited to function as a generic term for these phenomena.

### Defense motives and selective exposure

Health information can be threatening in various ways. For example, it may implicate that a health condition is present, or it may suggest a necessity of changing beloved everyday routines to maintain one’s health. Different defense motives may be triggered by different kinds of threats. In this context, Knobloch-Westerwick et al. (2013) Klicken oder tippen Sie hier, um Text einzugeben. introduce the term of self-defending motivation, which implies discrediting, ignoring, and avoiding information that (potentially) implies a threat to one’s health and physical wellbeing. For example, fear-appeal information suggesting an increased risk of developing cancer tends to be avoided by smokers—a classic example of selective exposure triggered by self-defending motivation. Empirically, health threats seem to be a strong driver of self-defending motivation, as is evidenced by a study by Greving et al. (2015), in which they showed that Internet search behavior is positively biased when there is an experimentally induced health threat. More specifically, after a fake diagnosis on the intolerance of a food additive, participants selected more positive links (e.g., that the intolerance also protects against diabetes) and less negative links (e.g., that the intolerance leads to a weakened immune defense) on a fictitious search engine results page (compared to a control condition with no health threat). While the exact theoretical mechanisms behind such effects are still unclear, they are in line with the notion of positive illusions (e.g., “unrealistically positive self-evaluations, exaggerated perceptions of control or mastery, and unrealistic optimism”; Taylor and Brown, 1988, p. 193), which are caused by a set of filters in the cognitive system that lead to individuals discarding or devaluating threatening information.

While the defense motives described above are specific for the health context, more general motives for selective exposure may play a role in health information seeking behavior, too. For example, one may selectively search for, and select information to confirm one’s opinion or expectation about a specific topic (Hart et al., 2009), or one may try to confirm one’s specific self-image as a way of self-affirmation (Munro and Stansbury, 2009). In line with this is the motivation to devaluate and downplay information that disconfirms opposing attitudes and opinions. These different motives may, in addition to health-related self-defending, lead to biased approaches to health information seeking. According to Hart et al. (2009), such motives fulfill a specific goal that is not related to finding out the facts and approaching the “truth,” but to protecting an intact self-image (Hart et al., 2009). Hart et al. (2009) argue that the psychological process behind this is cognitive dissonance (Festinger, 1957), a negative affective state that arises when external information is not in line with prior conceptions. More specifically, Hart et al. (2009) argue that “after people commit to an attitude, belief, or decision, they gather supportive information and neglect unsupportive information to avoid or eliminate the unpleasant state of post decisional conflict known as cognitive dissonance” (p. 556).

One crucial similarity can be identified in all of these different motives: They strive to protect parts of the self, be it the self-image, attitudes, and opinions (general motives), or the physical integrity (health-specific motives), as a consequence of a potential (health) threat and as a precondition for biased information seeking and/or appraisal (Munro and Stansbury, 2009; van ‘t Riet and Ruiter, 2013). Threat, however, is highly subjective and dependent on one’s perceived risk. For example, leaflets suggesting an increased risk for lung cancer in smokers do not imply a threat for non-smokers. Therefore, non-smokers would not have any motivation to discredit or ignore the leaflets, while smokers, on the other hand, may well try to actively disregard them. Thus, a threat can be regarded as a necessary precondition for selective exposure to information in health contexts. Therefore, perceived risk for a certain disease should be considered as a principal basis to appraise health information as threatening or not. In this line of reasoning, the higher the perceived risk, the higher should be the perceived threat and thus, a greater bias in information seeking should occur as various defense motives are activated.

However, taking “risk” into account as a precursor for selective exposure requires a differentiated look at the concept of risk. While perceived risk represents a potential precondition to perceiving a threat, suggested risk (i.e., by an information leaflet) must also be considered. A suggested risk implies that a certain individual characteristic like the body mass index (BMI), for example, is suggested to be associated with an increased risk of suffering from a health impairment (e.g., in an information leaflet on high BMI as a risk factor for cardiovascular disease). Depending on your individual BMI, this message might thus involve a threat (if your BMI is high) or not (if your BMI is low). Moreover, you may or may not have perceived a high risk for cardiovascular diseases in the first place. Hence, with suggested as well as perceived risk taken into account, several scenarios that may or may not trigger defense motives (and selective exposure) are conceivable. In fact, combining perceived and suggested risk (or risk feedback) leads to four possible combinations in individuals who are confronted with health information: *perceived risk* (low or high) crossed with *risk feedback* (suggested risk or no suggested risk).

### The present study

The present study aims to investigate the effects of defense motives on selective exposure to health information when a threat is induced *via* risk feedback—depending on the individual’s perceived risk. Based on our aforementioned theoretical considerations, we distinguish between the following two types of defense motives: First, the general motive to defend one’s opinion and attitudes by approaching confirming information and avoiding disconfirming information (see Hart et al., 2009), which we label “opinion-defending motive,” and, second, the more (health-)specific motive to maintain or defend a positive view of one’s health (Taylor and Brown, 1988; Greving and Sassenberg, 2015), which we label “health-defending motive.” Based on prior research, we argue that the opinion-defending motive is likely triggered by information that contradicts one’s opinion (Hart et al., 2009; Knobloch-Westerwick et al., 2013) whereas the health-defending motive should be triggered by information that suggests a health risk (Greving et al., 2015).

To put it bluntly, we consider the opinion-defending motive to be about being right, whereas the health-defending motive may be more about feeling healthy. Of course, everyone wants to be healthy *and* right—but in everyday life, a multitude of cases are conceivable where we are confronted with information that threatens either one or both of these motives. For example, the tendency to engage in selective exposure following a confrontation with information suggesting a health threat (which would trigger the health-defending motive) may be additionally boosted when this information is not in line with one’s opinion about one’s health status (which would trigger the opinion-defending motive). In contrast to this example where opinion-defending and health-defending motives are consistent, they may, however, also be dissonant. For example, imagine a person who believes that his diet is rather unhealthy. If this person receives information on the health-damaging effects of this diet, the person’s opinion is supported by the external information—even though the information itself is threatening to the person’s physical integrity. This congruence between the external information and the person’s opinion, in turn, may possibly buffer the effects of the health-defending motive that would usually lead to selective exposure to information denying the diet’s health risks. However, although generally acknowledged as two central precursors of a biased search for information, both types of defense motives have—to our knowledge— never been considered in one study simultaneously, let alone in the context of health information seeking. This is puzzling given the potential for a complex interplay between both motives, and corresponding experimental research may help us better understand the psychological dynamics that underlie selective exposure to health information.

**FIGURE 1:**
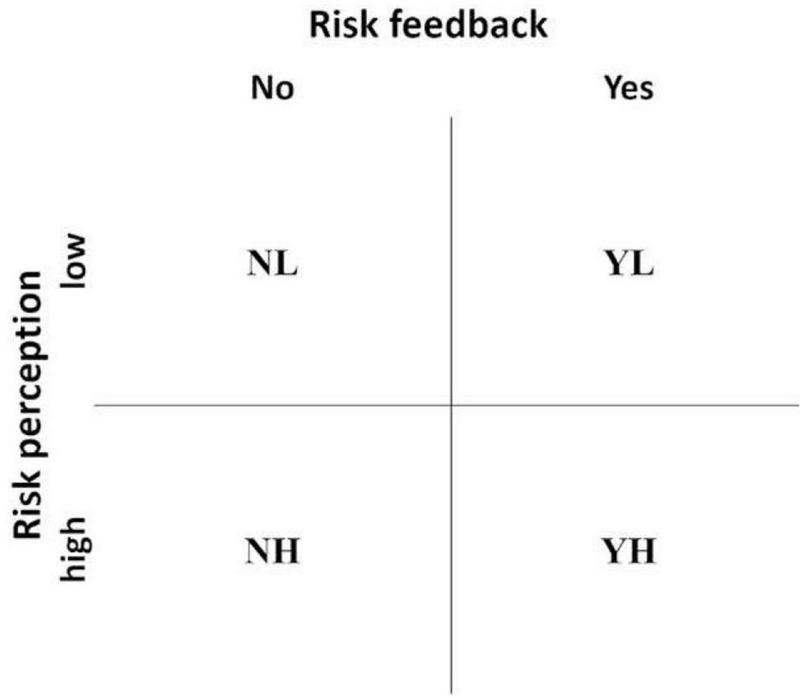
Overview of the experimental design.

For this reason, we applied a 2 × 2 design with one experimental factor “risk feedback” (suggested risk vs. no suggested risk) and one quasi-experimental factor “perceived risk” (high vs. low). With this, we tested the notion that feedback of a higher health risk (threat to self in the form of health/physical integrity; Knobloch-Westerwick et al., 2013) and feedback mismatching the self-assessed health risk (threat to self in the form of opinion or attitude; Hart et al., 2009) leads to selective exposure to health information. Crossing the two factors results in four different groups, each of which implies different conditions for showing selective exposure. The first group (*no* risk feedback and *low* risk perception = NL; see Figure 1) is characterized by the absence of an experimentally suggested risk and consists of participants who perceive themselves at low risk. Thus, in this group, there is an accordance between self-assessment and risk feedback, which is why the opinion-defending motive may not be triggered. The health-defending motive should not play a role either, as no risk feedback is given. No risk feedback is also given in another group (NH), which is, however, characterized by risk self-assessment (high risk) not corresponding to the given feedback (no risk). In this case, an opinion-defending motive would be conceivable since potentially long-established beliefs about the self are challenged, and the participants want to protect their own beliefs. The two other groups, in contrast, received risk feedback. In one of these two groups (risk feedback: *yes*, perceived risk: *high*; YH), the reported risk corresponds to one’s own perception, which is why the opinion-defending motive has no relevance. However, for the protection of one’s own physical integrity, as a reaction to the risk feedback, the health-defending motive may be relevant. While the health-defending motive maintains relevance in the last group (YL), the opinion-defending motive also becomes relevant. This group receives risk feedback, although individuals in this group perceive a rather low risk for themselves. Therefore, a conflict between risk self-assessment and risk feedback arises, which is the precondition for the opinion-defending motive. An overview of the four resulting groups can be found in Figure 1.

The study, including research design, study hypotheses, and statistical analyses, was preregistered at PsychArchives before data collection (Wedderhoff et al., 2019).

The dependent variables (DVs) are (1) the amount of selective exposure to information which suggests no risk in an information selection task on a fictitious Google results page, and (2) the quality ratings of every piece of information at participants’ disposal. Based on this, six hypotheses were formulated, one for each main effect of the two factors on each of the two dependent measures for selective exposure, and respectively, one for the interaction between the two factors. To induce the perception of a health threat in an experimental study, a scenario that is realistic, relevant, and understandable is essential. We opted to suggest an increased risk for developing heart disease caused by a specific degree of achievement motivation, which we had measured beforehand. This ensures a certain level of comprehensibility: The background is understandable and credible while, at the same time, purely fictitious (without the participants being aware of it). Moreover, from an ethical standpoint, an experimental manipulation based on the suggestion of a risk is not as problematic as a more direct induction of a health threat (e.g., by means of a fake medical exam suggesting that participants indeed *have* a health condition). The suggested risk may trigger both defensive motives. First, it may be a threat to physical integrity. Second, it poses a threat to participants’ self-image as it may contradict, depending on the experimental condition, their opinion about the individual risk (i.e., perceived risk). This leads to the following hypotheses:

*Hypothesis 1^1^: We expect a main effect of the perceived heart disease risk on selective exposure to information that suggests no risk: In the higher perceived risk conditions (YH and NH), selective exposure to information suggesting no risk will be stronger compared to the lower perceived risk conditions (YL and NL).*

*Hypothesis 2: We expect a main effect of the perceived heart disease risk on mean quality rating of information that suggests a risk: In the higher perceived risk conditions (YH and NH), the average quality rating of information suggesting a risk is lower compared to the lower perceived risk conditions (YL and NL).*

The psychological mechanism we expect to be behind these first two hypotheses is the health-defending motive. We expect that individuals who perceive themselves at a higher health risk generally strive for soothing or reassuring information, as a health threat is associated with a preferential processing of positive information (Greving et al., 2015; see section “Defense motives and selective exposure”). It should be noted that depending on the experimental group, the opinion-defending motive may well reduce these effects since searching for reassuring information counters the opinion-defending motive in individuals who perceive themselves at higher risk. Nevertheless, we argue that the opinion-defending motive is mainly triggered by external feedback (e.g., risk feedback), since such feedback constitutes a strong incentive to *defend* one’s opinion. Therefore, we argue that the health-defending motive should trump the opinion-defending motive when risk feedback is kept constant across conditions (as it is when analyzing main effects). Hence, overall, we expect a higher amount of selective exposure in the groups which perceive themselves at a higher heart disease risk (NH and YH) compared to the groups perceiving a lower risk (NL and YL).

*Hypothesis 3: We expect a main effect of the risk feedback on selective exposure to information that suggests no risk: In the conditions with risk feedback (YH and YL), selective exposure to information suggesting no risk will be stronger compared to the conditions with “no risk” feedback (NH and NL).*

*Hypothesis 4: We expect a main effect of the risk feedback on mean quality rating of information that suggests a risk: In the conditions with risk feedback (YH and YL), the average quality rating of information suggesting a risk is lower compared to conditions with “no risk” feedback (NH and NL).*

Again, the main diver behind these hypothesized effects is the health-defending motive. Even if it is in line with one’s opinion (YH group), risk feedback implies a threat to one’s physical integrity, which is why it should lead to stronger selective exposure compared to “no risk” feedback. In addition, external risk feedback constitutes a strong driver of selective exposure if this feedback is not in line with one’s opinion (YL group) because of the opinion-defending motive. This is because such a constellation gives an incentive to defend one’s opinion against an “attack” from the outside. Overall, we therefore expect a higher amount of selective exposure in the groups which receive “high risk” feedback (YH and YL) compared to the groups which receive “no risk” feedback (NH and NL).

*Hypothesis 5^2^: We expect an interaction effect between the perceived and the suggested risk of heart disease in different forms for each of the four conditions on selective exposure: Given that individuals with a low self-perceived risk who receive risk feedback should be most motivated to reject threatening information (i.e., because both motives are triggered), we expect that the direction of the main effect of self-perceived risk specified in Hypothesis 1 will reverse in individuals who are given risk feedback.*

*Hypothesis 6: We expect an interaction effect between the perceived and the suggested risk of heart disease in different forms for each of the four conditions on quality ratings. Given that individuals with a low self-perceived risk who receive risk feedback should be most motivated to reject threatening information, we expect that the direction of the main effect of self-perceived risk specified in Hypothesis 2 will reverse in individuals who are given risk feedback.*

These two interaction hypotheses are based on our expectations regarding the combined effects of the opinion-defending and the health-defending motive. In the case of risk feedback, more selective exposure should arise with decreasing self-perceived risk since a discrepancy between risk feedback and self-perceived risk likely prompts an opinion-defending motive (e.g., Hart et al., 2009), possibly through mechanisms such as cognitive dissonance. Additionally, risk feedback is likely to directly prompt a health-defending motive (e.g., Greving et al., 2015) in order to protect a healthy self-image (cf. positive illusions in section “Defense motives and selective exposure”). In this case, the opinion-defending and the health-defending motive thus act in concert. In contrast, if there is no risk feedback, both motives will become less and less important with decreasing self-perceived risk since this implies an increasing consistency between risk feedback and self-perceived risk (thus reducing the opinion-defending motive), and since there is no prompting of the health-defending motive *via* threatening information. We therefore expect the main effects specified in Hypotheses 1 and 2 (e.g., increased selective exposure with increased risk perceptions) to reverse when individuals are given risk feedback.

## Materials and methods

### Sample

To determine the sample size, we conducted a power analysis in GPower 3.1 (Faul et al., 2009). With power set to 0.80 and alpha to 0.05, a sample size of *N* = 787 is required to detect a small effect (*f* = 0.10) in a 2 × 2 ANOVA (numerator *df* = 1) when testing for main effects and interactions. We therefore aimed for a sample size of 800 participants (see preregistration; Wedderhoff et al., 2019). Overall, 847 German-speaking participants, aged between 30 and 65 years and with no medical history of heart disease, participated in the study. Eighty-four participants showed conspicuous response patterns. More specifically, *n* = 44 participants took less than 1,140 s to complete the study (which was less than half the median of the processing time), *n* = 36 participants did not respond to the DV, and *n* = 4 participants stated that they chose the eight snippets “at random” when asked to justify their responses on the DV (see below) in a free-text field at the end of the study^3^. These *n* = 84 participants were removed from the analysis, which resulted in a final sample of *N* = 763 (52.2% women; *M*_*age*_ = 51.17, *SD*_*age*_ = 10.42). The distribution of educational attainment levels was representative of Germany’s population. Age distribution was slightly skewed to the left, meaning that older participants were slightly more frequent than younger participants, thus also approximating the age distribution in the German general population. Considering the restricted age range of our sample (30–65 years) as specified in our inclusion criteria (see preregistration), this variable was not normally distributed. The sample was recruited through a panel, administered by a professional agency, and data collection was performed solely online.

### Procedure and materials

Ethical approval for the study was granted by the ethics committee of the German Psychological Society (DGPs). After completing an informed consent form and a check on whether the inclusion criteria were met, participants were told that current research is investigating how to explain the relationship between achievement motivation and heart disease. This was followed by an explanation that the study ties in and investigates how achievement motivation is distributed among the population and how people assess their personal risk of heart disease.

After this introduction, a number of covariates (i.e., potential moderators) were measured. Health information literacy (HIL) was assessed by a slightly adapted version of the Health Information Literacy Knowledge Test (HILK) (Mayer et al., 2018), and self-efficacy was measured by the Self-Efficacy Scale for Information Searching Behavior (Behm, 2018), using an instruction adapted to the search for health information. Additionally, for potential exploratory analyses, behavioral inhibition and behavioral approach system sensitivity (Carver and White, 1994) were assessed by a short-form of the ARES (Action Regulating Emotion Systems) scales (Hartig and Moosbrugger, 2003). Furthermore, a self-report instrument for the assessment of emotion-specific regulation skills (SEK-ES) (Ebert et al., 2014) was administered. To control whether the threat induction worked, the Positive and Negative Affect Schedule (PANAS) (Breyer and Bluemke, 2016) was applied before and after the induction, which would allow detecting potential affective changes. Next, the quasi-experimental factor “perceived risk” was measured by a self-developed single item (“*My risk of developing heart disease in the next 5 years*…”) with six response levels (1 = “*…is much lower compared to other people my age*” to 6 = “*…is much higher compared to other people my age*”). Participants reported a mean perceived risk of *M* = 3.09 (*SD* = 1.11), and a visual inspection revealed a normal distribution of the corresponding variable. Before the statistical analyses, the variable was median-split (median = 3), resulting in *n* = 430 participants in the low perceived risk group and *n* = 273 participants in the high perceived risk group. Finally, dispositional achievement motivation was assessed by the subscale “achievement motivation” of a German instrument measuring occupation-related personality variables, the “Bochumer Inventar zur berufsbezogenen Persönlichkeitsbeschreibung” (BIP; Hossiep and Krüger, 2012).

After completing these questionnaires and tests, a 50-s loading screen was presented along with the explanation that the inputs are processed, analyzed, and compared with a norm sample. This was to ensure a higher fidelity of the upcoming threat intervention. The participants were then randomly assigned to one of two conditions of the experimental factor “risk feedback,” which should induce a threat or no threat. Every participant’s real score and result of the BIP were displayed as well as the notion if it was higher or lower than average. This statement was combined with a text indicating a higher risk or indicating no risk for developing heart disease (depending on the experimental condition), which also included a reference to a fictitious research report that makes this assumption. Besides the PANAS, three self-constructed items were presented as an additional manipulation check, which assessed subjective feelings of threat and the corresponding information need (e.g., “*I find the information disturbing*” and “*I need more information on the subject*”) with five response options each (1 = “*Strongly disagree*” to 5 = “*Strongly agree*”). A mean score (variable *perception of threat*) was calculated before the statistical analyses; scale reliability was high with a Cronbach’s Alpha of α = 0.90.

Finally, participants completed a selection task to assess the DV selective exposure. The task is a variation of the task used by Adams et al. (2018) Klicken oder tippen Sie hier, um Text einzugeben. and was framed as an opportunity to obtain additional information about the relationship between heart disease and achievement motivation. They were presented with a (fictitious) Google results page including 16 search results drawing on a combination of the words “achievement motivation” and “heart disease,” from which they were asked to select eight results for further research. At the same time, they were asked to rate each search result concerning the quality of the information it provides (values from 1 to 6, with 6 corresponding to the highest quality). The search results included a title and short text snippets and were as realistic as possible in length and wording as well as in visual appearance, thus mimicking an actual Google page. The results differed in that they suggested either an increased or a reduced risk for the respective participant’s development of heart disease and, furthermore, whether they were serious (e.g., scientific articles, universities, public submissions) or dubious sources (e.g., yellow press, individual reports). They represented the best selection from a twice as large pool of snippets, which were checked for credibility and comprehensibility in a preliminary pilot study (*N* = 56). Using the data gathered from this task, our DV were computed. First, we calculated a selective exposure score by subtracting the number of selected results suggesting a risk from the number of selected results suggesting no risk. Since participants had to choose eight results, this results in a score ranging from –8 (all selected results suggest a risk) to +8 (all selected results suggest no risk). A score of 0 suggests a balanced selection of snippets, as it indicates that four snippets of each kind had been selected. Regarding the quality ratings of the different search results, the average quality rating of snippets suggesting a risk constituted the first quality rating DV (quality rating DV 1), and the average quality rating of snippets suggesting no risk constituted the second quality rating DV (quality rating DV 2).

**FIGURE 2:**
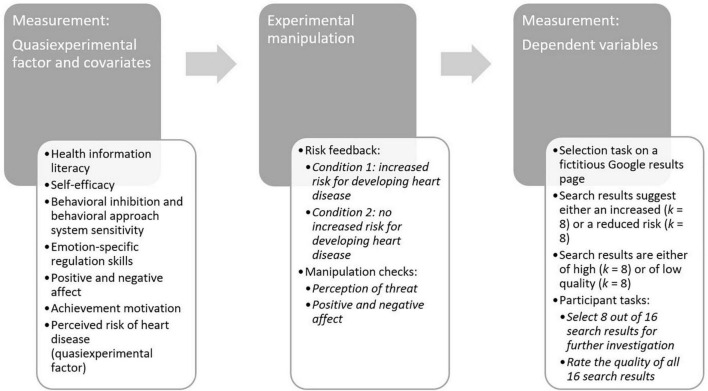
Overview of the study procedures.

After completion of the task, participants were asked to rate the perceived authenticity of the snippets and were presented with the final page of the survey containing a comprehensive debriefing. An overview of the study procedures can be found in Figure 2.

## Results

### Preliminary analyses

Table 1 reports the descriptive statistics and intercorrelations of the study variables. To test whether the manipulation of induced risk through the feedback of potential risk for heart disease worked, the mean score of the variables for the perception of threat was investigated. The score ranged between 1 (“*no threat*”) and 5 (“*high threat*”). The two groups, “no risk feedback” and “risk feedback,” differed significantly in their perception of threat (*t* = −11.53, *df* = 735, *p* < 0.001). The average score for the “no risk feedback” group was *M* = 1.55, with 58% of the participants having a score of 1. In the “risk feedback” group, the average score was *M* = 2.40, with 28% of the participants having a score of 1. Concerning the PANAS scores, only the “risk feedback” group showed a significant reduction of positive affect between the two measurement points (*t* = 6.18, *df* = 414, *p* < 0.001, *M*_*T*1_ = 3.10, *SD*_*T*1_ = 0.80 *M*_*T*2_ = 2.95, *SD*_*T*2_ = 0.82). Therefore, it seems that the induction of risk for the corresponding condition was successful. To additionally investigate whether the effects of the threat induction varied over participants depending on their perceived risk, we conducted a two-factorial ANOVA with the independent variables *risk feedback* and *perceived risk* (median-split; see above) and the DV *perception of threat*. While the main effect of risk feedback remained significant and of large effect size (*F*[1,759] = 141.077, *p* < 0.001, η^2^ = 0.157), we also found a small but significant main effect of perceived risk (*F*[1,759] = 21.506, *p* < 0.001, η^2^ = 0.028) and a small but significant interaction between both factors (*F*[1,759] = 7.824, *p* < 0.01, η^2^ = 0.010). This interaction, according to a visual inspection of the corresponding plots, suggested that individuals with high perceived risk were more susceptible to the risk feedback (in terms of an increased perception of threat) compared to individuals with low perceived risk. Finally, all prerequisites (independence of groups, normal distribution of the dependent variable [DV], and homogeneity) for further analyses were tested and were fulfilled.

**TABLE 1:** Descriptive statistics and intercorrelations of study variables.

Variable	*M*	*SD*	1	2	3	4	5	6
(1) Age	51.17	10.42						
(2) HIL	0.69	0.13	−0.094**					
(3) Emotion Regulation	3.55	0.77	0.001	0.259**				
(4) Self-Perceived Risk	3.09	1.11	0.026	–0.028	−0.166**			
(5) Selective Exposure	0.37	2.93	−0.100**	0.077*	0.071	–0.017		
(6) Quality Rating of Snippets Suggesting No Risk	3.18	0.69	0.018	–0.007	0.035	0.062	0.001	
(7) Quality Rating of Snippets Suggesting a Risk	3.17	0.74	0.007	0.016	0.066	0.068	0.018	0.633**

*N* = 763.

HIL, Health Information Literacy.

*p < 0.05; **p < 0.01.

### Confirmatory analyses

To examine the impact of perceived risk (high vs. low) and risk-feedback (yes vs. no) on respondents’ selective exposure, univariate analyses of variance were conducted with these two factors.

#### Effects on selective exposure

**FIGURE 3:**
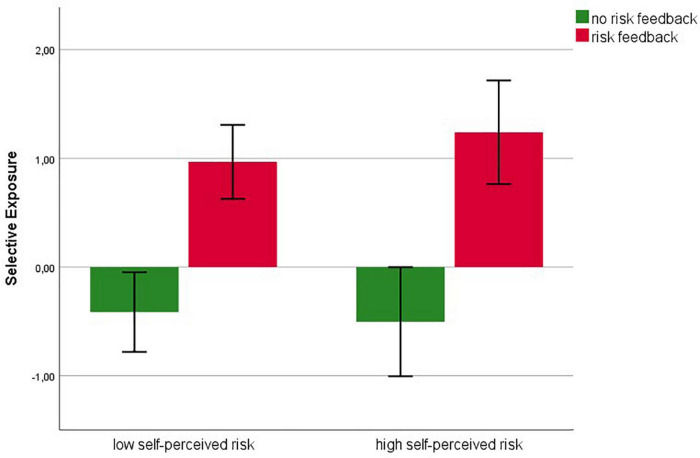
Descriptive overview of selective exposure scores across experimental conditions, including error bars with 95% confidence intervals.

A descriptive overview of selective exposure scores across experimental conditions, including error bars with 95% confidence intervals, can be found in Figure 3. Effects on the selective exposure DV were tested in a two-factorial ANOVA with the independent variables *risk feedback* and *perceived risk* (median-split; see above). In this analysis, a main effect for risk feedback was found, with *F*(1,759) = 52.92, *p* < 0.001, η^2^ = 0.065. Examination of estimated marginal means indicated that participants with feedback of a higher risk selected more snippets that communicate no risk than participants with no risk feedback (*M*_*noRisk*_ = −0.45, *SE*_*noRisk*_ = 2.80 vs. *M*_*Risk*_ = 1.06, *SE*_*Risk*_ = 2.86), thus supporting hypothesis 3. Neither the hypothesized main effect of perceived risk (*F*[1,759] = 0.182, *p* = 0.67, η^2^ = 0.0002), nor the postulated interaction between perceived risk and risk feedback became significant (*F*[1,759] = 0.71, *p* = 0.40, η^2^ = 0.001). Hypotheses 1 and 5 thus were not confirmed.

#### Effects on quality rating

Effects on the two quality rating DVs were tested in two separate two-factorial ANOVAs with the independent variables risk feedback and perceived risk (median-split; see above). With regard to the average quality rating of snippets suggesting a risk (quality DV 1), results revealed no significant main effect for risk feedback (*F*[1,759] = 2.068, *p* = 0.15, η^2^ = 0.003), no significant main effect for perceived risk (*F*[1,759] = 1.203, *p* = 0.27, η^2^ = 0.002), and no significant interaction between both factors (*F*[1,759] = 0.245, *p* = 0.62, η^2^ = 0.0003). With regard to the average quality rating of snippets suggesting no risk (quality DV 2), results again revealed no significant main effect for risk feedback (*F*[1,759] = 0.554, *p* = 0.46, η^2^ = 0.001), no significant main effect for perceived risk (*F*[1,759] = 2.672, *p* = 0.10, η^2^ = 0.004), and no significant interaction between both factors (*F*[1,759] = 1.193, *p* = 0.28, η^2^ = 0.002). Thus, hypotheses 2, 4, and 6 were not confirmed.

### Exploratory analyses

Exploratory analyses aimed at gaining further insight into factors that moderate how the two independent factors (perceived risk and risk feedback) influence the DVs of selective exposure and quality assessment. In this regard, two influential and often mentioned constructs come into mind: HIL (Meppelink et al., 2019) and emotion regulation (Das, 2012; van ‘t Riet and Ruiter, 2013). As we had found a significant main effect of risk feedback on selective exposure, we investigated the corresponding interactions for the risk feedback factor. Hayes’ PROCESS macro (Hayes, 2013) was used to test for the potential moderation of both HIL and emotion regulation on the relation between risk feedback and selective exposure and quality rating (see Table 2). In addition, we investigated whether participant age may have affected our confirmatory hypotheses tests.

**TABLE 2:** PROCESS results for moderator analyses with selective exposure as outcome.

Model	Variable	*R* ^2^	Coefficient	*t*	*p*
1		0.31			0.00
	Constant		0.80	0.99	0.32
	(X) Risk Feedback		–3.13	–2.90	0.00
	(W) HIL		–1.79	–1.57	0.12
	Interaction		6.70	4.38	0.00
2		0.28			0.00
	Constant		–0.65	–0.91	0.36
	(X) Risk Feedback		–0.21	–0.21	0.83
	(W) Emotion Regulation		0.06	0.29	0.77
	Interaction		0.49	1.83	0.06

Results are from concurrent regression analyses. The resulting coefficients are unstandardized B parameters. X, independent variable; W, moderator; HIL, Health Information Literacy.

#### Health information literacy

Health information literacy is defined by the Medical Library Association as “*the set of abilities needed to recognize health information need; identify likely information sources and use them to retrieve relevant information; assess the quality of the information; and analyze, understand, and use the information to make good health decisions*” (Shipman et al., 2009). Although the notion “set of abilities” is a bit unspecific, HIL is necessarily involved in every health information gathering process. Hence, HIL should also play an important role in the phenomenon of selective exposure, as it supports searching and selecting specific information. Yet it remains unclear exactly how HIL influences the incidence of selective exposure. Two possibilities are conceivable: (1) A more pronounced HIL promotes a balanced search, as all relevant information is considered and used for good health decisions; or (2) with higher HIL, the well-developed ability to search and evaluate information enables a stronger selection of information according to the objectives of the defensive motives (Meppelink et al., 2019). Empirically, we found a significant interaction between risk feedback and HIL (*b* = 6.70; *p* < 0.001; *R*^2^ change when adding the moderator = 0.023, see Table 2) as predictors of selective exposure, while the direct effect of risk feedback also remained significant. Closer inspection of this interaction showed that with increasing HIL, selective exposure became increasingly stronger when participants were confronted with risk feedback compared to no risk feedback. Interaction probing using the Johnson–Neyman technique (see Table 3) revealed that this was significant for all HIL values below the cut-off value of 0.265 (with 1.18% of cases scoring lower than this value) and for all HIL values above the cut-off value of 0.554 (with 87.55% of cases scoring higher than this value). For quality ratings, no significant results were found.

**TABLE 3:** PROCESS results for interaction probing according to the Johnson–Neyman technique with HILK as moderator and selective exposure as outcome.

HILK score	Effect	*SE*	*t*	*p*
0.0714	–26.498	0.9753	–27.170	0.007
0.2552	–14.178	0.7026	–20.180	0.044
0.2653	–13.503	0.6879	–19.631	0.050
0.3012	–11.098	0.6356	–17.461	0.081
0.5309	0.4301	0.3226	13.332	0.183
0.5536	0.582	0.2965	19.631	0.050
0.5769	0.7381	0.2716	27.176	0.007
0.8066	22.780	0.2656	85.785	<0.001

HILK, Health Information Literacy Knowledge Test.

#### Emotion regulation

Emotion regulation is the ability to leave or alter an emotional state (Baumann and Kuhl, 2002; Koole, 2009). In a state where a health threat is present, the discussed defensive motives aim to minimize negative feelings through reassuring or confirming information (Hart et al., 2009), which may be in contrast to a comprehensive search. In previous studies, a negative affective state was found to predict health information seeking behavior (Hastall and Wagner, 2018). A neutral or less negative affective state should therefore promote a more balanced and comprehensive search. In relation to this, it is important, for an adequate search while facing a threat, that one has a certain ability to regulate potentially negative emotions that may arise (Das, 2012). Accordingly, van ‘t Riet and Ruiter (2013) Klicken oder tippen Sie hier, um Text einzugeben. state that emotion regulation ability affects the exposure to various kinds of health-promoting information. Hence, we also assume a moderating effect on the relation of the regarded factors with selective exposure and quality rating. As negative emotions have a higher relevance for defense motives (Jonas et al., 2016), we only considered emotion regulation for negative emotions. However, only a marginally significant effect on the interaction of risk feedback and emotion regulation to predict selective exposure was found (*b* = 0.49; *p* = 0.06; *R*^2^ change when adding the moderator = 0.004; see Table 2), and the main effect of risk feedback that was found before disappeared when including the interaction term. While these results must be considered with some caution because the interaction (narrowly) missed the *p* < 0.05 criterion, a closer inspection revealed that the participants in the risk feedback condition tended to select more information which denies a threat (i.e., higher selective exposure) with increasing emotion regulation ability. In contrast, participants in the no risk feedback condition seemed not to be affected by different levels of emotion regulation ability, as they did not differ in their selective exposure results.

#### Age

Older individuals often have more health problems and often feel more threatened by disease compared to younger persons (e.g., Szabo et al., 2020). In addition, there is evidence for age-related biases with regard to information processing (e.g., Teuscher, 2009; Carstensen and DeLiema, 2018). For this reason, we investigated whether our findings may vary with regard to different age groups. Since the age variable in our dataset was not normally distributed, we decided to conduct a median-split (median = 53 years) and calculate three separate three-factorial ANOVAs (i.e., using our three DVs, see above). These analyses were identical to our confirmatory hypotheses tests, but additionally included the age variable as well as two two-way interactions between age and risk feedback respectively age and perceived risk, and a three-way interaction between age, risk feedback, and perceived risk. Similar to our confirmatory analyses, we found no significant main effects or interactions with regard to the two quality DVs (all *p* > 0.066). However, with regard to the selective exposure DV, we found a significant interaction between age and perceived risk (*F*[1,755] = 4.003, *p* = 0.046, η^2^ = 0.005), as well as a significant three-way interaction between age, perceived risk, and risk feedback (*F*[1,755] = 9.135, *p* = 0.003, η^2^ = 0.012). In addition, the pattern of results regarding risk feedback was very similar to our confirmatory analyses (see above). To investigate this further, we conducted the exact same analyses as we did when testing our confirmatory hypotheses (see above), but this time separately for younger and older participants. These analyses revealed a marginally significant main effect of perceived risk on selective exposure in older adults (*F*[1,388] = 3.836, *p* = 0.051, η^2^ = 0.010), but not in younger adults (*F*[1,367] = 0.924, *p* = 0.337, η^2^ = 0.003). This effect was in the expected direction (i.e., older adults with a higher perceived risk showed more selective exposure compared to older adults with a lower perceived risk), thus providing partial and tentative support for hypothesis H1. In addition, the two-way interaction between perceived risk and risk feedback was significant in younger adults (*F*[1,367] = 7.252, *p* = 0.007, η^2^ = 0.019), but not in older adults (*F*[1,388] = 2.132, *p* = 0.145, η^2^ = 0.005). However, the specific pattern of the interaction in younger adults was contrary to our expectations because, at least on a descriptive level, the main effect of perceived risk was negative in the no risk feedback condition (i.e., more perceived risk led to less selective exposure) and positive in the risk feedback condition (i.e., more perceived risk led to more selective exposure). Hypothesis 5 is not supported.

## Discussion

The present paper aimed to gain further insight into the effects of two defense motives—a self-confirming and a self-defending motive—on respondents’ selective exposure to health information. Overall, our findings indicate that a suggested health risk influences selective exposure to health information, while a self-perceived risk seems to have no significant effect in this context. As predicted in our preregistration, we found that risk feedback leads to stronger bias toward the preference of information which denies the risk: Receiving feedback which suggests a potential health risk shifted task performance from a rather balanced selection of snippets to a biased selection of snippets that deny a particular risk. Furthermore, it seems that in the context of one’s own health, the motivation to defend one’s self-image from a threat (which we labeled the health-defending motive; see above) is superior to the motivation to confirm one’s opinion (i.e., the opinion-defending motive). This is because, in the condition of no risk feedback, respondents showed no significant bias in either direction—even in the case of a high perceived risk. While it should be noted that we found some tentative and exploratory evidence for a corresponding bias in older participants, it generally seems that participants neither confirmed their own risk perception when they saw themselves as being at higher risk, nor did they deny a risk and therefore confirm the “no risk” feedback. Together with the significant effects of the risk feedback, this can be interpreted as an indication that in such an essential and potentially existentially relevant context as the health context, coping with a health threat has a higher implicit value than the need to confirm one’s opinion.

This is in line with other findings from the field of coping research that, in general, suggest that there is a stronger bias when individuals are in a negative emotional state, which may be more strongly triggered by an unexpected and immediate risk feedback compared to self-perceptions that have probably been present for a long time (Johnson and Case, 2012). Moreover, selective exposure seems to be stronger when the focus lies more on losses instead of gains (Rothermund et al., 2008). In this case, the threat of physical integrity can be seen as a loss (losing health status), while the defense of one’s own opinion is mentally represented rather as a gain (one wants to be proven correct) and thus, is less susceptible to bias.

In this sense, an opinion-defending motive seems less likely to come into effect in the case of health threats and the associated autonomous search for information. Rather, it is conceivable that potential risks and threats are avoided *via* the self-directed (biased) choice of information channels, a process which is described in the theory of counter-regulation (Rothermund, 2011). According to this theory, negative states, elicited, for example, through health-threatening information, are “counteracted” by actively turning toward positive (e.g., reassuring or unrelated) information. Our explorative findings also partly support this claim: Participants with a higher ability to regulate their negative emotions showed a more biased selection toward positive information, which may provide reassurance thereby allowing them to downregulate their negative feelings.

Our results regarding the moderating effect of HIL further support these assumptions. In fact, higher HIL led to a stronger selective exposure. This means that with a higher HIL, less balanced information is considered, which at first may seem counter-intuitive. In general, HIL is associated with positive health outcomes (Berkman et al., 2011; Hirvonen et al., 2016), which initially does not seem to match with an unbalanced consideration of relevant health information. However, because the performance test that we used to measure HIL primarily addresses the abilities to search, acquire, and evaluate suitable sources and health information (according to the definition of HIL), this effect suggests that basic abilities of information processing may be “misused” in the present case to meet one’s needs and motives. In this regard, Meppelink et al. (2019) also showed a biased selection of messages that were in line with their own beliefs concerning vaccination (regardless of the line of argumentation against or in favor of) for participants with higher health literacy. They also showed a higher prevalence of biased perceptions of message convincingness for people with higher health literacy. Similarly, a study by Drummond and Fischhoff (2017) found that science literacy was associated with greater political and religious polarization, which is, according to the authors, “consistent with … the motivated reasoning account, by which more knowledgeable individuals are more adept at interpreting evidence in support of their preferred conclusions” (p. 9590). Accordingly, future research should dive into what may be considered the “dark side of information literacy,” and interventions on HIL should consider extending their aims to include the aspect of a balanced search.

Furthermore, the non-significant results for perceived risk indicate a need for further research. As stated before, the opinion-defending motive may not be as important when one’s own health is threatened. Nevertheless, our experiment shows an overall tendency toward biased information selection when it comes to health topics, and, furthermore, we concede that our claims that the opinion-defending motive would be less important are based on the interpretation of non-significant results. To disentangle the effects of the two defense motives in future studies, some adjustments to the paradigm and evaluation task are advisable. In contrast to the currently used cover story, it could be beneficial to use a more ambivalent and controversial health topic where the own opinion is held at high stake. At the same time, the cover story should not induce such a large threat in order to prevent triggering *only* the self-defending motive—at least for a portion of the participants. Such topics could include, for example, the efficacy of homeopathic drugs or vaccine hesitancy (Meppelink et al., 2019). This makes it possible to develop scenarios in which the two motives are activated both separately and simultaneously (e.g., in different experimental groups). In the case of homeopathy, for example, risk feedback based on a homeopathic “assessment” may be perceived as much more threatening to physical well-being by homeopathy supporters. In contrast, homeopathy skeptics would supposedly rather doubt the content and see their own convictions threatened.

Another potential explanation of why only one of our hypotheses was confirmed could be ascribed to the nature of the selection task. With eight to-be-selected snippets out of a total of 16 snippets, the resulting cognitive load when performing the task might have been excessive, which could have almost automatically led to a rather balanced selection. A significant reduction of the number of snippets should force a selection on the basis of the currently active motive(s). However, a disadvantage of this procedure would be that the lower number of selected snippets leads to a lower variance in the DV because possible resulting values are restricted. Our initial idea was that the relatively high number of eight selected snippets would result in more detailed differences in the extent of selective exposure, depending on the independent variables and moderators. Another solution to this problem was recently implemented by Kerwer et al. (2021). In their study, only four snippets were presented at a time, from which one had to be selected for further reading. This was done four times so that a total number of 16 snippets were presented while simultaneously reducing cognitive load. Two final limitations that should be considered when interpreting our findings pertain to our risk feedback manipulation. First, it is rather likely that individuals with a higher perceived risk or with a poorer health status (e.g., high BMI) are more susceptible to a threat induction. We tested this empirically in an exploratory analysis on the threat perception variable from the manipulation check, and indeed found support for the notion that individuals with higher perceived risk are more susceptible to risk feedback. However, these effects were rather small, while the effect size on the experimental manipulation itself (i.e., the induction of threat through risk feedback) was large. Nevertheless, the issue warrants caution when interpreting our results. Second, related to this issue, an inconsistency between perceived risk and risk feedback may not only lead to the triggering of an opinion-defending motive, but also (or instead) to a desire to resolve the inconsistency (e.g., by changing one’s opinion), or it may simply lead to doubt in the experiment itself. Future research should strive to straighten out which motives come into effect in which case, and also try to discern the cognitive processes (e.g., dissonance) behind the emergence of different motives – for example by using techniques such as think-aloud protocols.

### Implications

Some rather ambivalent implications can be gleaned from the findings of the present study. In line with Sassenberg and Greving (2016) Klicken oder tippen Sie hier, um Text einzugeben., our results suggest that an autonomous selection of information may help patients react to a health threat *via* consulting reassuring information about their health. One could argue that this is a positive implication in the sense that it may help them to develop a more positive view of their body and make them feel better. However, the findings also implicate that a suggested health threat leads to a bias in information selection. This might be because, as we have discussed, a suggested risk increases negative affective states like anxiety, which trigger defense motives to feel better and/or reassured. This is also in line with previous research that states that the likelihood of a unilateral selection of positive information is higher when a negative affective state is present, which is also referred to as “counter-regulation” (Rothermund et al., 2008; Schwager and Rothermund, 2013, 2014). Research on health message perception and on the effects of fear appeals in health-promoting information also supports our findings and points to further implications (van ‘t Riet and Ruiter, 2013; Ruiter et al., 2014). In fact, health information that emphasizes individual risk factors does not automatically cause the recipient to implement appropriate behavior to reduce the risk (i.e., giving up smoking). On the contrary, such information often evokes defensive cognitive and behavioral reactions, such as ignoring, denying, or downplaying it (van ‘t Riet and Ruiter, 2013). In contrast, messages that, besides pointing to a significant health threat, suggest ways to diminish the threat and enhance the recipients’ self-efficacy seem to be more effective with regard to changes in health behavior (Schwarzer, 2008; Ruiter et al., 2014). Positive affect and a substantial amount of confidence to be able to deal with the threat thus seem to be essential in order to avoid a bias toward positive information and to select information in a less biased manner (Das, 2012; Ruiter et al., 2014). It is therefore conceivable that, as a consequence, individuals who are in a negative affective state because they have been threatened by risk suggesting information have a biased (positive) picture of their own health, resulting from biased information retrieval in the past. This poses the danger that they underestimate potential health risks and do not consider necessary interventions. In this respect, Sassenberg and Greving (2016) Klicken oder tippen Sie hier, um Text einzugeben. also refer to the risk of a potential negative impact on the doctor–patient relationship, as patients could be too confident about their health status, and become impervious to reasonable arguments that point in another direction.

## Conclusion

Our study provides evidence for selective exposure and bias in health information seeking. In the presence of an externally suggested threat to their health, individuals tend to reassure themselves and therefore show a selective exposure to positive information. This may also override a potential motivation to defend one’s own opinion when it is in conflict with the reassuring information. However, further research and adjustments to the information selection task are required to investigate these rather tentative conclusions.

What is certain, however, is that an independent search for health information is increasingly deemed necessary and seems to be implicated by modern health care systems in terms of the promotion of patient empowerment and informed decision making. Nevertheless, the wide availability of health-related information to the general population also creates new risks for imbalanced information acquisition and use. Selective exposure might help patients to reassure themselves and cope with their emotional states, but it may also lead to an incorrect assessment of their individual health (risk) status.

## Data availability statement

The original contributions presented in the study are publicly available. This data can be found here: doi: 10.23668/PSYCHARCHIVES.2770.

## Ethics statement

The studies involving human participants were reviewed and approved by Ethics Committee of the German Psychological Society (DGPs). The patients/participants provided their written informed consent to participate in this study.

## Author contributions

OW, AC, and TR conceived and planned the study. OW and AC conducted the data collection, analyzed the data, and prepared a first draft of the manuscript. TR reviewed and revised the manuscript and prepared it for publication. All authors contributed to the article and approved the submitted version.

## Footnotes

1 As a minor deviation from the preregistration, all hypotheses have been slightly adapted for clarity and precision.

2 It should be noted that in the preregistration, the interactions are specified with regard to expected mean differences across groups, and thus in greater detail.

3 While we consider these deviations as major protocol deviations justifying the elimination of cases, the preregistration mentioned more than 50% missing data per case as the only example for a major protocol deviation (which did not occur in our dataset). As a further deviation from our preregistration, we did not compute Mahalanobis distances to screen for multivariate outliers given the high complexity of our data structure and the fact that it is rather unusual to do so.
